# Protease Activated Receptor Signaling Is Required for African Trypanosome Traversal of Human Brain Microvascular Endothelial Cells

**DOI:** 10.1371/journal.pntd.0000479

**Published:** 2009-07-21

**Authors:** Dennis J. Grab, Jose C. Garcia-Garcia, Olga V. Nikolskaia, Yuri V. Kim, Amanda Brown, Carlos A. Pardo, Yongqing Zhang, Kevin G. Becker, Brenda A. Wilson, Ana Paula C. de A. Lima, Julio Scharfstein, J. Stephen Dumler

**Affiliations:** 1 Department of Pediatrics, The Johns Hopkins University School of Medicine, Baltimore, Maryland, United States of America; 2 Department of Pathology, The Johns Hopkins University School of Medicine, Baltimore, Maryland, United States of America; 3 Department of Neurology, The Johns Hopkins University School of Medicine, Baltimore, Maryland, United States of America; 4 Gene Expression and Genomics Unit, National Institute on Aging, National Institutes of Health, Baltimore, Maryland, United States of America; 5 Department of Microbiology, University of Illinois at Urbana-Champaign, Urbana, Illinois, United States of America; 6 Instituto de Biofísica Carlos Chagas Filho, Universidade Federal do Rio de Janeiro, Rio de Janeiro, Brazil; New York University School of Medicine, United States of America

## Abstract

**Background:**

Using human brain microvascular endothelial cells (HBMECs) as an *in vitro* model for how African trypanosomes cross the human blood-brain barrier (BBB) we recently reported that the parasites cross the BBB by generating calcium activation signals in HBMECs through the activity of parasite cysteine proteases, particularly cathepsin L (brucipain). In the current study, we examined the possible role of a class of protease stimulated HBMEC G protein coupled receptors (GPCRs) known as protease activated receptors (PARs) that might be implicated in calcium signaling by African trypanosomes.

**Methodology/Principal Findings:**

Using RNA interference (RNAi) we found that in vitro PAR-2 gene (*F2RL1*) expression in HBMEC monolayers could be reduced by over 95%. We also found that the ability of *Trypanosoma brucei rhodesiense* to cross *F2RL1*-silenced HBMEC monolayers was reduced (39%–49%) and that HBMECs silenced for *F2RL1* maintained control levels of barrier function in the presence of the parasite. Consistent with the role of PAR-2, we found that HBMEC barrier function was also maintained after blockade of Gα_q_ with *Pasteurella multocida* toxin (PMT). PAR-2 signaling has been shown in other systems to have neuroinflammatory and neuroprotective roles and our data implicate a role for proteases (i.e. brucipain) and PAR-2 in African trypanosome/HBMEC interactions. Using gene-profiling methods to interrogate candidate HBMEC pathways specifically triggered by brucipain, several pathways that potentially link some pathophysiologic processes associated with CNS HAT were identified.

**Conclusions/Significance:**

Together, the data support a role, in part, for GPCRs as molecular targets for parasite proteases that lead to the activation of Gα_q_-mediated calcium signaling. The consequence of these events is predicted to be increased permeability of the BBB to parasite transmigration and the initiation of neuroinflammation, events precursory to CNS disease.

## Author Summary

Human African trypanosomiasis, or sleeping sickness, occurs when single-cell trypanosome protozoan parasites spread from the blood to brain over the blood-brain barrier (BBB). This barrier is composed of brain microvascular endothelial cells (BMECs) especially designed to keep pathogens out. Safe drugs for treating sleeping sickness are lacking and alternative treatments are urgently required. Using our human BMEC BBB model, we previously found that a parasite protease, brucipain, induced calcium activation signals that allowed this barrier to open up to parasite crossing. Because human BMECs express protease-activated receptors (PARs) that trigger calcium signals in BMECs, we hypothesized a functional link between parasite brucipain and BMEC PARs. Utilizing RNA interference to block the production of one type of PAR called PAR-2, we hindered the ability of trypanosomes to both open up and cross human BMECs. Using gene-profiling methods to interrogate candidate BMEC pathways specifically triggered by brucipain, several pathways that potentially link brain inflammatory processes were identified, a finding congruent with the known role of PAR-2 as a mediator of inflammation. Overall, our data support a role for brucipain and BMEC PARs in trypanosome BBB transmigration, and as potential triggers for brain inflammation associated with the disease.

## Introduction

Human African trypanosomiasis (HAT), commonly called sleeping sickness, is a vector-borne disease for which death is inevitable if the patient is untreated [1],[2],[3]. HAT is caused by two subspecies of African trypanosomes, *Trypanosoma brucei rhodesiense* and *T. b. gambiense* causing East African and West African sleeping sickness, respectively. In classical late stage HAT (stage 2), the parasites invade the central nervous system (CNS) and the infected individuals suffer from progressive neurologic deterioration with concomitant psychiatric disorders, sleep disturbances, stupor, and coma. The role of the parasites in the pathogenesis of CNS lesions is not completely understood [4].

Using an *in vitro* model of the blood-brain barrier (BBB) consisting of human brain microvascular endothelial cells (HBMEC), we showed that human infective *T. b. rhodesiense* have a high potential for transendothelial migration, while animal infective *T. b. brucei* cross inefficiently [5]. We initially proposed that African trypanosome associated protease(s) could mediate the process of parasite traversal across the BBB [6]. *In vitro* studies utilizing both cysteine protease inhibitors and RNA interference (RNAi) have identified a key role for the *T. brucei* Clan CA (papain) family of cysteine proteases in the lifecycle of *T. b. brucei* [7],[8]. The *T. brucei* genome encodes two distinct Clan CA cysteine proteases. Brucipain (aka trypanopain-*Tb*, rhodesain) is a cathepsin L-like protease responsible for the bulk of protease activity in the organism [7]. *T. brucei* cathepsin B (*Tb*CatB) has activity that is upregulated in the bloodstream form (BSF) of the parasites [7]. Remarkably, it was found that the ability of the parasites to cross HBMECs through the generation of PLC/PKC mediated Ca^2+^ activation signals correlated with levels of brucipain activity [9],[10]. RNAi studies investigating the roles played by *Tb*CatB and brucipain in the pathogenesis of *T. b. brucei in vivo* also suggested brucipain possibly facilitates parasite entry into the CNS [11].

Cysteine proteases can activate a class of G protein coupled receptors (GPCR) known as protease activated receptors (PARs). In neutrophils and oral epithelial cells PAR activation by gingipain, a cysteine protease of *Porphyromonas gingivalis*, leads to calcium signaling and IL-6 production [12],[13],[14]. HBMEC express all 4 protease activated receptors (PARs), and activation of PAR-1 and PAR-2 has been shown to trigger calcium mediated BMEC transmembrane signaling [15]. Since we observed activating signals (increases in [Ca^2+^]_i_ and phospholipase C activation) during the interaction of African trypanosomes with HBMECs [5],[9],[10], we hypothesized that the activation of PARs by the parasite could also play a role in increasing HBMEC permeability, enabling subsequent crossing [4]. We now show that African trypanosome traversal across the human BBB requires, at least in part, the participation of a PAR-2-mediated calcium signaling pathway(s).

## Materials and Methods

### Chemicals

N-methyl-Pip-F-homoF-vinyl sulfonyl phenyl (K11777) [16],[17], an irreversible inhibitor of *Trypanosoma cruzi* Clan A cysteine protease cruzipain [17] was a gift from Dr. James McKerrow (University of California at San Francisco).

### Purification of recombinant *Pasteurella multocida* toxin

Recombinant *Pasteurella multocida* toxin (PMT) was cloned, expressed, purified, quantified, and tested for biological activity as previously described [18]. The toxin samples were stored at −80°C until use.

### HBMEC and trypanosomes

Primary HBMECs (≤ passage 13) were maintained as previously described [5],[9],[15]. The bloodstream form (BSF) *T. b. rhodesiense* used was originally obtained from the CSF from a Kenyan patient with sleeping sickness [5],[9],[10]. This parasite was formerly classified as *T. b. gambiense* IL1852, but has been reclassified as *T. b. rhodesiense* IL1852 based on the presence of the SRA gene [10]. The bloodstream form (BSF) trypanosomes were maintained in culture in HMI-9 [19]. For the microarray study, to inactivate brucipain activity, the parasites were pretreated for 30 min with 5 µM of the cathepsin-L inhibitor K11777 The parasites were then washed with medium to remove excess inhibitor prior to incubation with HBMEC monolayers [9],[10].

### Electrical cell-substrate impedance sensing for real-time transendothelial electrical resistance measurement and cell signaling

The elevated transendothelial electrical resistance (TEER) and the lower paracellular permeability of the brain microvasculature is a characteristic feature that distinguishes it from non-brain endothelium. Measurement of TEER is a one of the most straightforward methods to access the barrier tightness using *in vitro* models [20]. Electrical Cell-Substrate Impedance Sensing (ECIS) gathers TEER data as an electrical method for assessing barrier function that detect changes in endothelial cell shape in real-time [21]. The Model 1600R ECIS system (Applied Biophysics) [22],[23],[24] was used to measure HBMEC TEER changes in real-time during exposure to African trypanosomes and their secreted products. HBMECs were grown on collagen-coated 8-well single (8W1E) or multiple (8W10E^+^) gold electrode ECIS arrays until confluent. While changes in relative TEER recorded by the single and multiple arrays were similar, absolute TEER values differ. Steady state TEER >10,000 ohms and >1,000 ohms were used for the 8W1E and 8W10E^+^ arrays respectively (Applied Biophysics). The multiple electrode arrays in which HBMEC resistances are >1,000 ohms [4],[5],[25],[26] record the activities of more cells over a larger region of the substrate. However, the 10-fold lower capacitance of the 8W1E array leads to increased resistances (more than 10 times that of the multiple electrode arrays) and a higher signal to noise ratio (ECIS 1600R instruction manual). Changes in resistance of HBMEC monolayers were monitored every 80 sec in response to experimental variables. For the PMT experiments, HBMECs were simultaneously incubated with PMT (30 ng/ml) or pretreated with the toxin for 90 min then washed with fresh medium prior to incubation with parasites.

### HBMEC gene silencing by RNAi and analysis by laser capture microdissection (LCM)

In single cell studies, when stimulated with PAR-2 agonists strong Ca^2+^ signals are induced ([15] in >60% of HBMEC (YV Kim, unpublished) suggesting a role for PAR-2 in parasite HBMEC traversal. We silenced the *F2RL1* expression by co-transfecting a pre-designed siRNA for *F2RL1* and a GFP-expressing plasmid into subconfluent HBMECs using Lipofectamine 2000 and standard protocols. A matched negative control siRNA (Ambion) was used as control. To determine the efficiency of gene silencing, HBMECs were grown on 35 mm LCM dishes (PALM) prior to RNAi silencing. Using laser capture microdissection (LCM), 15 individual GFP-HBMECs that expressed GFP in the *F2RL1-*silenced and control siRNA cultures were collected. The GFP-HBMECs were marked and catapulted using the following settings on the P.A.L.M. Microlaser (Bernied, Germany): energy-cut of 60; energy-lpc of 86; focus-cut of 75 and focus-lpc of 92 [27]. The RNA extracted using Ambion's RNAqueous®-Micro kit was then amplified using Ambion's MessageAmp™II kit. qRT-PCR was done using pre-designed *F2RL1* primers (Invitrogen) and the data were normalized to *ACTB* transcripts.

### Co-incubation of HBMECs with trypanosomes

HBMECs grown to confluency in Transwell inserts, ECIS arrays or 6-well microtiter plates were incubated at 37°C in 5% CO_2_ in Experimental Medium (HMI-9 and Medium 199 mixed 1∶1) containing 10% FBS [5],[9],[10]. HBMEC were incubated in triplicate with BSF *T. b. rhodesiense* IL1852 (5×10^5^ to 1×10^6^/ml) under the indicated study conditions.

### Transcriptome microarray analysis

Samples from 2 independent experiments containing duplicate sets of infected (wild type or K11777 pretreated trypanosomes) and uninfected HBMECs were rapidly dissociated with trypsin/EDTA, than washed. Total cellular RNA was isolated using the RNAeasy kit (QIAGEN) following the manufacturer's instructions. Purified RNA was treated with RNase-free DNase to remove contaminating genomic DNA. The integrity of RNA transcripts was verified by electrophoresis through denaturing agarose-formaldehyde gels followed by ethidium bromide staining [28].

cDNAs were radiolabeled with ^32^P α-dCTP Isoblue (ICN) using SuperScript II Reverse Transcriptase (Invitrogen). Unbound label was separated using a Biospin P-30 spin column (Bio-Rad). Each cDNA probe was adjusted to 10^6^ cpm/mL and hybridized to separate nylon MGC-1 microarrays [29] at 68°C overnight in Microhyb hybridization solution (Research Genetics). The MGC-1 microarray represents 9,600 different human gene features including those encoding cytokines and other immunological regulatory proteins such as chemokines, growth factors, and cellular receptors [29]. Membranes were washed three times in 2×SSC (1×SSC is 0.15 M NaCl plus 0.015 M sodium citrate)-1% SDS for 30 min at 68°C and twice in 0.1× SSC-0.5% SDS for 30 min at 68°C. Membranes were exposed overnight and scanned on a Molecular Dynamics STORM phosphoimager set to 50-micron resolution. mRNA expression levels were analyzed by scanning densitometry using ArrayPro imaging software. Differential patterns of gene expression were assessed by preparing RNA from both uninfected control HBMEC or HBMEC that were co-incubated with wild-type (WT) *T. b. rhodesiense* IL1852 or *T. b. rhodesiense* K11777 inhibited for brucipain activity for 3 and 6 hours. These cDNAs were hybridized in parallel to pairs of identical microarrays.

Raw microarray data were subjected to Z normalization and tested for significant changes as previously described [30]. Genes were determined to be differentially expressed after calculating the Z ratio, which indicates the fold-difference between experimental groups, and false discovery rate (fdr), which controls for the expected proportion of falsely rejected hypotheses. Individual genes with p value ≤0.05, absolute value of Z ratio>1.5 and fdr<0.3 were considered significantly changed. Hierarchical cluster method with complete algorithm and K-mean cluster were employed to identify clustering within groups. Array data for each experimental condition were hierarchically clustered with the DIANE 6.0 software in JMP 6.0 environment and R programs. Pilot studies showed no signal detected when trypanosome cDNA was hybridized to the microarrays.

Probability scores for each network or functional Parametric analysis of gene set enrichment (PAGE ) [31] gene set analysis was performed by using the PAGE algorithm and PERL/R code with MYSQL database. The archive of gene sets used with this algorithm for this analysis is from the Molecular Signatures Database (MSigDB) (http://www.broad.mit.edu/gsea/msigdb/genesets.jsp?collection=CP) [32]. Significance of functions and pathways was calculated using the z-test between each function group genes and the genes in the whole sample. The p-value was calculated by comparing the number of user-specified genes of interest participating in a given function or pathway relative to the total number of occurrences of these genes in all functional/pathway annotations stored in the knowledge base. The Enrichment score, which is called pathway z-scores, were calculated by the difference of the mean z-ratio of the selected function groups with the mean of z-ratio of the whole sample genes to represent the pathway change altitude.

Array data from infected samples are presented as relative changes to uninfected controls in mRNA expression following normalization of gene signals to total signal and levels of housekeeping gene mRNA to ensure analysis of equivalent amounts of RNA. This approach facilitated the direct comparison of mRNA levels between control and the parasite-treated HBMEC.

## Results/Discussion

### The role of PAR-2 in trypanosome transmigration across HBMECs

**Figure 1:**
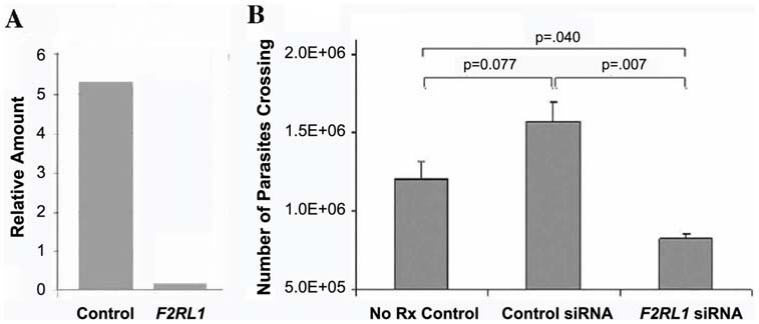
*T. b. rhodesiense* transmigration across HBMEC silenced for *F2RL1* expression by RNAi. Using laser capture microdissection 15 individual HBMEC expressing GFP in the *PAR2*-silenced and control siRNA cultures were collected and the RNA extracted. (A) Based on qRT-PCR using pre-designed *F2RL1* primers and normalized to *ACTB* (β-actin transcripts), PAR-2 gene expression was reduced by over 95%. (B) *T. b. rhodesiense* IL1852 was incubated for 16h in triplicate with HBMEC monolayers silenced for PAR-2 expression (*F2RL1* siRNA), with a matched scrambled siRNA control (control siRNA) construct, or with untreated HBMEC (no Rx control) and examined parasite transmigration.

**Figure 2:**
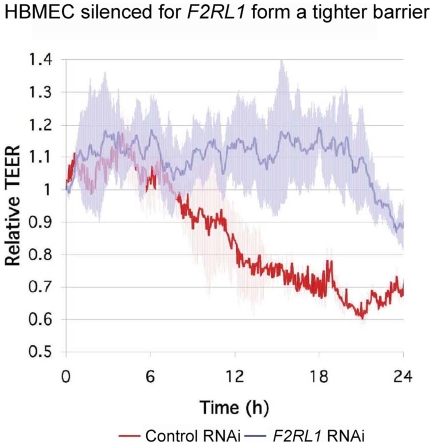
Real-time TEER changes in HBMEC silenced for PAR-2 gene expression. *F2RL1* RNAi transfected HBMEC grown in 8W1E ECIS chambers were incubated overnight with *T. b. rhodesiense* IL1852. The changes in TEER relative to matched HBMEC control containing scrambled siRNA construct (red line) and HBMEC silenced for *F2RL1* (blue line) in the presence of parasites are shown.

After silencing of *F2RL1* by RNAi, based on qRT-PCR targeting *F2RL1* transcripts normalized to *ACTB* (β-actin transcripts), we found that PAR-2 expression was reduced by over 95% (Fig 1A) in LCM-isolated HBMECs. To verify whether the ability of *T. b. rhodesiense* to cross HBMECs requires host cell PAR-2 signaling, we incubated *T. b. rhodesiense* IL1852 for 16h with HBMEC monolayers silenced for *F2RL1* expression (*F2RL1* siRNA), transfected with a matched scrambled siRNA control (control siRNA), or with untreated HBMECs (no Rx control) (Fig 1B) and examined parasite transmigration. There was no statistical difference in parasite transmigration between the 2 control samples: in medium alone and or medium with control siRNA (p = 0.077; 2-tail Student t-test). Considering a 95% reduction in *F2RL1* expression in the ∼60% of the HBMEC that express PAR-2, significant differences were observed between *F2RL1*-silenced HBMECs compared to both control conditions: 39% inhibition (p = 0.040) versus untreated HBMECs (no Rx control) and 49% inhibition (p = 0.007) versus control siRNA-treated HBMECs. In the absence of PAR-2-induced signaling, we predicted that *F2RL1*-silenced primary HBMECs would maintain a tighter barrier upon stimulation by *T. b. rhodesiense*. ECIS was used to monitor real-time TEER changes in HBMEC monolayer integrity. *F2RL1* RNAi-transfected HBMECs grown in ECIS chambers were incubated overnight with *T. b. rhodesiense* IL1852 (1.2×10^6^/mL). Fig 2 shows that unlike the matched HBMEC scrambled siRNA control (red line), HBMECs silenced for PAR-2 maintained control TEER levels (about 10,000 ohms in the 8W1E arrays used) (blue line) for at least 20 hours even in the presence of a high parasite load.

### The Gα-specific toxin from *Pasteurella multocida* blocks trypanosome-induced changes in TEER in HBMEC

**Figure 3:**
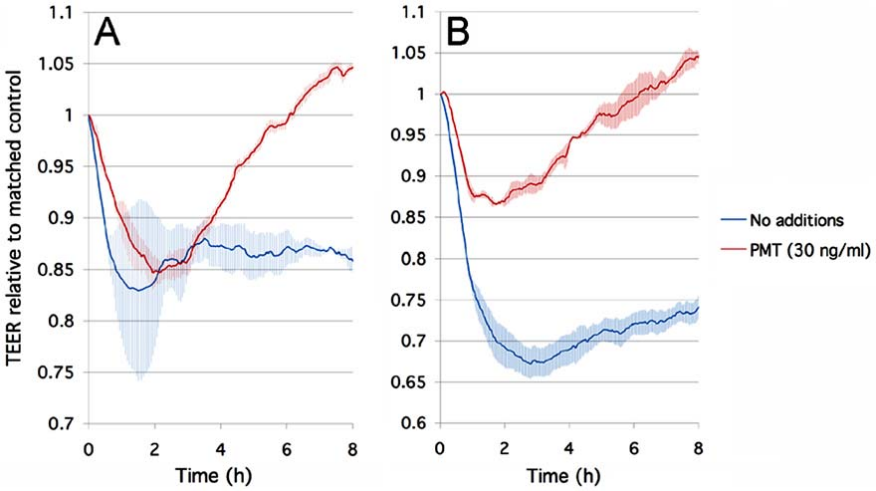
PMT from *Pasteurella multocida* blocks *T. b. rhodesiense* induced changes in HBMEC TEER. HBMEC grown in 8W10E+ ECIS chambers were incubated with *T. b. rhodesiense* IL1852. Shown are the changes in real-time TEER measured by ECIS. A) HBMECs incubated with *T. b. rhodesiense* IL1852 in the absence (blue line) or continuous presence of PMT (30 ng/ml) (red line). B) HBMECs incubated with *T. b. rhodesiense* IL1852 was incubated with HBMECs untreated (blue line) or pretreated with PMT (30 ng/ml) (red line). The data are represented as the average change in TEER±EM (n = 3). The changes in TEER are represented as the average change in TEER±SEM (n = 2).

PARs, including PAR-2, are GPCRs known to mediate their cellular effects through the activation of Gα_q/11_, Gα_12/13_ and Gα_i_βγ signaling pathways [33],[34],[35],[36]. The protein toxin from *Pasteurella multocida* (PMT) has been shown to target Gα_q_ [37],[38],[39], Gα_12/13_ [40] and Gα_i_ [41] heterotrimeric G proteins in eukaryotic cells. PMT potentiates Gα_q_ protein-mediated GPCR responses to ligands by primarily activating phospholipase C (i.e. PLC-β1, 3, 4) [37],[38],[39],[42]. Accordingly, this leads to calcium mobilization and activation of PKCs, as well as activation of mitogenic pathways, including MAPK (ERK1/2, p38) activation [39],[42],[43]. PMT enters cells via receptor-mediated endocytosis and acts intracellularly to activate Gα_q_ [37],[38],[39],[44]. This is subsequently followed by uncoupling of Gα_q_ signaling when cellular Gα_q_-mediated responses then become refractory to further stimulation [45],[46]. This also occurs when HBMECs are incubated with PMT. As shown in Fig. 3A, when incubated together with HBMECs and *T. b. rhodesiense,* PMT (30 ng/ml) initially does not inhibit the parasite-induced drop in TEER by ECIS. However, TEER increases above control levels with parasites after 2 hours, consistent with uncoupling of Gα_q_ signaling by PMT. When HBMECs are pretreated for 3h with PMT to allow for Gα_q_ uncoupling prior to trypanosome addition, the toxin clearly inhibited the ability of the parasites to compromise the HBMEC monolayers (Fig 3B). Since PAR-2 is a GPCR that can act via Gαq signaling, taken together, the data strongly suggest a role for PAR-2 and host Gαq-mediated calcium signaling in parasite interactions with the human BBB. While not yet tested would it be interesting to see the effect of PMT treatment on PAR-2 RNAi treated cells; i.e. are there additive effects of PMT treatment in these cells or is the PMT effect occluded by PAR-2 knockdown.

### Gene expression pathways in HBMEC in response to African trypanosomes lacking brucipain activity

PAR-2 signaling has been shown in other systems to have neuroinflammatory and neuroprotective roles [47],[48],[49],[50] and our data implicate a role for brucipain and PAR-2 in African trypanosome/HBMEC interactions. Therefore, we used transcription-profiling methods to interrogate candidate HBMEC pathways with particular attention paid to pathways that are specifically triggered by brucipain and that potentially link cellular processes to physiologic (i.e. CNS passage across the BBB) and pathophysiologic (neuroinflammation) processes associated with CNS HAT [4]. *T. b. rhodesiense* inhibited for brucipain activity via pretreatment with K11777 (a cell-permeable class-specific irreversible inhibitor of brucipain) are defective in crossing HBMECs [5],[9],[10],[16],[17]. We interrogated candidate HBMEC pathways whose expression was modulated by exposure to *T. b. rhodesiense* pretreated with the brucipain inhibitor K11777. Because the half-life of brucipain is not known, the experimental time frame was kept to 6 hours, the approximate doubling time of the parasite. This was done to minimize the potential problems in data interpretation because of the contribution of decreasing drug within the parasites that were doubling.

**Figure 4:**
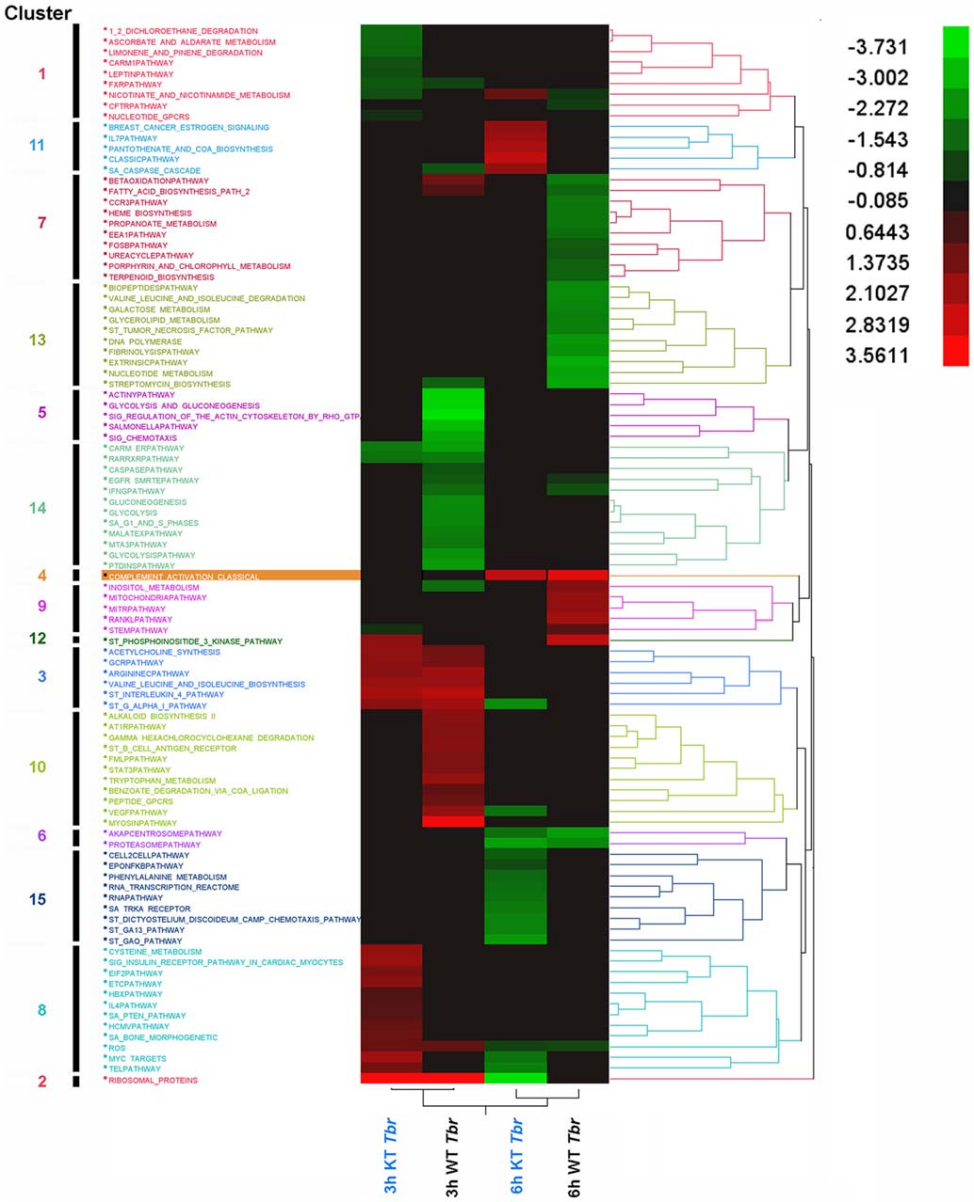
Pathway changes altered by *T. b. rhodesiense* inhibited for brucipain activity. Significantly regulated, functional pathway clusters were generated from wild-type (WT *Tbr*) *T. b. rhodesiense* or parasites K11777 inhibited for brucipain activity (KT *Tbr*) gene sets using PAGE gene set analysis. Pathways that were significantly up-regulated (red) or down-regulated (green) are shown. Cluster group numbers are shown on the left, while the grading scale is in the upper right.

Gene set enrichment analysis of HBMEC based on the known canonical pathways showed that WT and modified African trypanosomes differentially altered the expression of genes represented in 99 pathways relative to the uninfected controls (Fig 4; Table 1). While the 99 pathways clustered into 15 groups according to their gene expression profiles, 28 pathways clustered into 4 unique cluster groups that were specifically expressed by HBMECs only after exposure to the K11777-pretreated trypanosomes (Table 1). A functional overview of the MSigDB gene sets was then done to categorize a small number of selected “gene families” whose members shared a common feature such as homology or biochemical activity, although not necessarily having common origins. Analysis of the gene functions within the HBMEC pathways showed 47 genes differentially expressed by HBMECs in response to trypanosome infection (Table 2). From this gene subset, 30 genes were expressed exclusively by HBMEC in response to brucipain-inhibited parasites (Tables 2 and 3). Of these, 30 genes functionally i) 3 encoded cytokines, ii) 16 encoded transcription factors, iii) 8 encoded kinases, iv) 3 encoded for translocated genes, and v) 4 encoded for oncogenes. Interestingly, HBMEC genes encoding for cell surface markers and tumor suppressors in response to wild-type trypanosomes, were not expressed by brucipain-inhibited parasites (Table 2).

**Table 1:** Gene expression in pathways altered only by *T. b. rhodesiense* inhibited for brucipain activity.

Time	Pathway Name	Cluster	Annotation	z-Score	
				3 h	6 h
**After 3 h**	1_2_DICHLOROETHANE_ DEGRADATION	1	[GenMAPP]	−1.55	n.s.
	ASCORBATE_AND_ALDARATE_ METABOLISM	1	[GenMAPP]	−1.55	n.s.
	CARM1_PATHWAY	1	The methyltransferase CARM1 interacts with transcription factors such as CBP/p300 and methylates histones H3 and H4. [BioCarta]	−1.10	n.s.
	LEPTIN PATHWAY	1	Leptin is a peptide secreted by adipose tissue that, in skeletal muscle, promotes fatty acid oxidation, decreases cells' lipid content, and promotes insulin sensitivity. [BioCarta]	−1.04	n.s.
	LIMONENE_AND_PINENE_ DEGRADATION	1	[GenMAPP]	−1.47	n.s.
	NUCLEOTIDE_GPCRS	1	[GenMAPP]	−0.49	n.s.
	CYSTEINE_METABOLISM	8	[GenMAPP]	1.89	n.s.
	EIF2_PATHWAY	8	Eukaryotic initiation factor 2 (EIF2) initiates translation by transferring Met-tRNA to the 40S ribosome in a GTP-dependent process. [BioCarta]	1.52	n.s.
	ETC_PATHWAY	8	Energy is extracted from carbohydrates via oxidation and transferred to the mitochondrial electron transport chain, which couples ATP synthesis to the reduction of oxygen to water. [BioCarta]	1.74	n.s.
	HBX_PATHWAY	8	Hbx is a hepatitis B protein that activates a number of transcription factors, possibly by inducing calcium release from the mitochondrion to the cytoplasm. [BioCarta]	0.83	n.s.
	HCMV_PATHWAY	8	Cytomegalovirus activates MAP kinase pathways in the host cell, inducing transcription of viral genes. [BioCarta]	1.18	n.s.
	IL4_PATHWAY	8	IL4 promotes Th2 cell differentiation via a heterodimeric receptor that activates Stat6/JAK and MAP kinase pathways. [BioCarta]	0.89	n.s.
	SA_BONE_MORPHOGENETIC	8	Bone morphogenetic protein binds to its receptor to induce ectopic bone formation and promote development of the viscera. [SigmaAldrich]	1.24	n.s.
	SA_PTEN_PATHWAY	8	PTEN is a tumor suppressor that dephosphorylates the lipid messenger phosphatidylinositol triphosphate. [SigmaAldrich]	0.90	n.s.
	SIG_INSULIN_RECEPTOR_ PATHWAY_IN_CARDIAC_ MYOCYTES	8	Genes related to the insulin receptor pathway. [SIGNALINGAlliance]	1.98	n.s.
**After 6 h**	BREAST_CANCER_ESTROGEN_SIGNALING	11	Genes preferentially expressed in breast cancers, especially those involved in estrogen-receptor-dependent signal transduction. [GEArray]	n.s.	1.82
	CLASSIC_PATHWAY	11	The classic complement pathway is initiated by antibodies and promotes phagocytosis and lysis of foreign cells as well as activating the inflammatory response. [BioCarta]	n.s.	2.62
	IL7_PATHWAY	11	IL7 is required for B and T cell development and proliferation and may contribute to activation of VDJ recombination. [BioCarta]	n.s.	2.05
	PANTOTHENATE_AND_COA_ BIOSYNTHESIS	11	[GenMAPP]	n.s.	2.27
	CELL_2_CELL PATHWAY	15	Epithelial cell adhesion proteins such as cadherins transduce signals into the cell via catenins, which alter cell shape and motility. [BioCarta]	n.s.	−1.31
	EPO_NFKB_PATHWAY	15	The cytokine erythropoietin (Epo) prevents stress-induced neuronal apoptosis by stimulating anti-apoptotic pathways through JAK2 kinase and NFkB. [BioCarta]	n.s.	−1.02
	PHENYLALANINE_ METABOLISM	15	[GenMAPP]	n.s.	−1.52
	RNA_TRANSCRIPTION_ REACTOME	15	[GenMAPP]	n.s.	−1.61
	RNA_PATHWAY	15	dsRNA-activated protein kinase phosphorylates elF2a, which generally inhibits translation, and activates NFkB to provoke inflammation. [BioCarta]	n.s.	−1.69
	SA_TRKA_RECEPTOR	15	The TrkA receptor binds nerve growth factor to activate MAP kinase pathways and promote cell growth. [SigmaAldrich]	n.s.	−1.84
	ST_DICTYOSTELIUM_ DISCOIDEUM_CAMP_ CHEMOTAXIS_PATHWAY	15	The fungus *Dictyostelium discoideum* is a model system for cytoskeletal organization during chemotaxis. [SIGNALING Transduction KE]	n.s.	−2.04
	ST_GA13_PATHWAY	15	G-alpha-13 influences the actin cytoskeleton and activates protein kinase D, PI3K, and Pyk2. [SIGNALING Transduction KE]	n.s.	−1.99
	ST_GAQ_PATHWAY	15	G-alpha-q activates phospholipase C, resulting in calcium influx and increasing protein kinase C activity. [SIGNALING Transduction KE]	n.s.	−2.45

These gene sets are canonical representations of a biological process compiled by domain experts. PAGE gene set analysis was performed using the DIANE 6.0 software using the PAGE algorithm. The archive of gene sets used with this algorithm for this analysis is from the MSigDB. Significance of functions and pathways was calculated using the right-tailed Fisher's Exact Test. The p-value was calculated by comparing the number of user-specified genes of interest participating in a given function or pathway relative to the total number of occurrences of these genes in all functional/pathway annotations stored in the knowledge base.

**n.s.: not significant**

**Table 2:** Functional overview of significant categorized MSigDB gene sets based on the pathways altered by both wild-type and *T. b. rhodesiense* inhibited for brucipain activity.

	Cytokines	Transcription Factors	Cell Surface Markers	Kinases	Translocated Genes	Oncogenes	Tumor Suppressors
**Tumor suppressors**		*TP53*					*CDKN2A, CFL1, PTEN, TP53*
**Oncogenes**				***CDK4,*** * RAF1*	***CCND1***	***CCND1, CDK4,*** * HRAS, * ***MDM2, PTPN1,*** * RAF1*	
**Translocated genes**		***NFKB2***	*TRFC*	***LCK***	***CCND1, LCK, NFKB2,*** * TRFC*		
**Kinases**				***ACTR2, CAMK2B, CDK2, CDK4, FYN, LCK, MAP2K2,*** * MAP3K3P,* ***PAK4,*** * RAF1, RPS6KA1, * ***RPS6KA2, RPS6KB2***			
**Cell surface Markers**			*CD38, TRFC*				
**Transcription factors**		***ERCC3, GATA3, GTF2F*** *1, HDAC1, * ***HMGB1,*** * KLF5, * ***MEF2A,*** * MEF2B, * ***MYOD1, NFATC3, NFKB2,*** * NFKBIB, * ***NFKBIE, NFYB,*** * NROB, * ***NR1H3, RELA,*** * STAT1, * ***TAF6, TAF9,*** * TP53*					
**Cytokines**	*IFNGR1, * ***IFNGR2, IL2RG, IL6***						

A functional overview of the MSigDB gene sets categorized into a small number of selected gene families whose members a common feature such as homology or biochemical activity. They do not necessarily have common origins. Annotation of pathway genes significantly expressed in HBMEC only in response to *T. b. rhodesiense* inhibited for brucipain activity are shown in **bold font.**

**Table 3:** Annotation of pathway genes significantly expressed in HBMEC only in response to *T. b. rhodesiense* inhibited for brucipain activity.

Gene Family [MSigDB]	Symbol	MsigDB Accession No.	Gene Names	GO Definitions	z-Ratio	
					3 h	6 h
cytokine	***IFNGR2***	BC003624	interferon gamma receptor 2 (interferon gamma transducer 1)	cell surface receptor linked signal transduction	**0.81**	n.s.
				integral to plasma membrane		
				antiviral response protein activity		
cytokine	***IL2RG***	BC014972	interleukin 2 receptor, gamma (severe combined immunodeficiency)	cell proliferation	n.s.	**1.12**
				integral to plasma membrane		
				interleukin-2 receptor activity		
cytokine	***IL6***	BC015511	interleukin 6 (interferon, beta 2)	acute-phase response	n.s.	**0.84**
				extracellular space		
				IL-6 receptor ligand activity		
kinase	***CAMK2B***	BC019070	calcium/calmodulin-dependent protein kinase (CaM kinase) II beta	protein amino acid phosphorylation	**0.80**	n.s.
				ATP binding activity		
kinase	***CDK2***	BC003065	cyclin-dependent kinase 2	G2/M transition of mitotic cell cycle	**0.75**	n.s.
				cytoplasm		
				ATP binding activity		
kinase	***MAP2K2***	BC000471	mitogen-activated protein kinase kinase 2	protein amino acid phosphorylation	**1.98**	n.s.
				extracellular		
				ATP binding activity		
kinase	***PAK4***	BC002921	p21(CDKN1A)-activated kinase 4	cell motility	**−1.25**	n.s.
				Golgi apparatus		
				ATP binding activity		
kinase	***RPS6KA2***	BC002363	ribosomal protein S6 kinase, 90kDa, polypeptide 2	protein amino acid phosphorylation	**1.36**	n.s.
				nucleus		
				ATP binding activity		
kinase	***RPS6KB2***	BC000094	ribosomal protein S6 kinase, 70kDa, polypeptide 2	protein amino acid phosphorylation	**1.46**	n.s.
				ATP binding activity		
kinase, translocated gene	***LCK***	BC013200	lymphocyte-specific protein tyrosine kinase	RAS protein signal transduction	n.s.	**1.65**
				membrane fraction		
				ATP binding activity		
oncogene	***MDM2***	BC009893	Mdm2, transformed 3T3 cell double minute 2, p53 binding protein (mouse)	negative regulation of cell proliferation	**−1.69**	n.s.
				nucleus		
				ligase activity		
oncogene	***PTPN1***	BC015660	protein tyrosine phosphatase, non-receptor type 1	protein amino acid dephosphorylation	**0.66**	n.s.
				cytoplasm		
				prenylated protein tyrosine phosphatase activity		
oncogene, kinase	***CDK4***	BC010153	cyclin-dependent kinase 4	G1/S transition of mitotic cell cycle	**1.11**	n.s.
				ATP binding activity		
oncogene, translocated gene	***CCND1***	BC014078	cyclin D1 (PRAD1: parathyroid adenomatosis 1)	G1/S transition of mitotic cell cycle	**−1.13**	n.s.
				cellular_component unknown		
transcription factor	***ACTR2***	BC014546	ARP2 actin-related protein 2 homolog (yeast)	cell motility	**−1.58**	n.s.
				Arp2/3 protein complex		
				structural constituent of cytoskeleton		
transcription factor	***ERCC3***	BC008820	excision repair cross-complementing rodent repair deficiency, complementation group 3 (xeroderma pigmentosum group B complementing)	regulation of transcription\, DNA-dependent nucleus ATP dependent DNA helicase activity	**−1.62**	n.s.
transcription factor	***FYN***	NM_002037	FYN oncogene related to SRC, FGR, YES	cell growth and/or maintenance ATP binding activity	**−0.36**	**0.61**
transcription factor	***GATA3***	BC006793	GATA binding protein 3	defense response (activates Th2 cytokine gene expression) nucleus transcription factor activity	**1.44**	n.s.
transcription factor	***GTF2F1***	BC000120	general transcription factor IIF, polypeptide 1, 74kDa	regulation of transcription\, DNA-dependent	n.s.	**0.77**
				transcription factor TFIIF complex		
				DNA binding activity		
transcription factor	***HMGB1***	BC003378	high-mobility group box 1	DNA unwinding	**0.97**	n.s.
				chromatin		
				single-stranded DNA binding activity		
transcription factor	***MEF2A***	BC013437	MADS box transcription enhancer factor 2, polypeptide A (myocyte enhancer factor 2A)	muscle development	**−1.34**	n.s.
				nucleus		
				transcription co-activator activity		
transcription factor	***MYOD1***	BC000353	myogenic factor 3	cell differentiation	**1.86**	n.s.
				nucleus		
				RNA polymerase II transcription factor activity\, enhancer binding		
transcription factor	***NFATC3***	BC001050	nuclear factor of activated T-cells, cytoplasmic, calcineurin-dependent 3	inflammatory response	**−0.90**	n.s.
				nucleus		
				transcription co-activator activity		
transcription factor	***NFKBIE***	BC011676	nuclear factor of kappa light polypeptide gene enhancer in B-cells inhibitor, epsilon	cytoplasm	**1.79**	n.s.
				transcription factor binding activity\, cytoplasmic sequestering		
transcription factor	***NFYB***	BC005316	nuclear transcription factor Y, beta	regulation of transcription\, DNA-dependent	**0.82**	n.s.
				nucleus		
				transcription factor activity		
transcription factor	***NR1H3***	BC008819	nuclear receptor subfamily 1, group H, member 3	regulation of transcription\, DNA-dependent	**−0.94**	n.s.
				nucleus		
				steroid hormone receptor activity		
transcription factor	***RELA***	BC014095	v-rel reticuloendotheliosis viral oncogene homolog A, nuclear factor of kappa light polypeptide gene enhancer in B-cells 3, p65 (avian)	anti-apoptosis	**1.40**	n.s.
				transcription factor complex		
				protein binding activity		
transcription factor	***TAF6***	BC018115	TAF6 RNA polymerase II, TATA box binding protein (TBP)-associated factor, 80kDa	regulation of transcription\, DNA-dependent	**1.18**	n.s.
				transcription factor TFIID complex		
				DNA binding activity		
transcription factor	***TAF9***	BC007349	TAF9 RNA polymerase II, TATA box binding protein (TBP)-associated factor, 32kDa	regulation of transcription\, DNA-dependent	**0.51**	n.s.
				transcription factor TFIID complex		
				DNA binding activity		
translocated gene, transcription factor	***NFKB2***	BC002844	nuclear factor of kappa light polypeptide gene enhancer in B-cells 2 (p49/p100)	cell growth and/or maintenance	**−2.37**	n.s.
				nucleus		
				transcription co-activator activity		

Raw microarray data were subjected to Z normalization and tested for significant changes. Genes were determined to be differentially expressed after calculating the Z ratio and fdr.

**n.s.: not significant**

Analysis of the HBMEC pathways specifically altered by the brucipain-inhibited parasites included those found only within down-regulated Clusters-1 and Cluster-15, or up-regulated Cluster-8 and Cluster-11. Cluster-1 consisted of 6 different pathways that had negative Z-score values (and were therefore positively effected by brucipain) by 3-hours, and that returned to control levels by 6h (Figure 4, Table 1). While roles for the metabolic/degradation pathways in Cluster-1 in HAT are not clear, the data suggest brucipain alters the CARM1 and Leptin pathways. Leptin is a protein hormone typically produced by adipose tissue that is known to regulate appetite via binding to the leptin receptor (LEPR) in the hypothalamus after crossing the BBB [51],[52]. It has been suggested that compromised leptin transport into the CNS resulting in low leptin levels in the hippocampus could lead to cognitive deficits [53]. There is evidence that the secretion of photoperiodic hormones such as melatonin is inversely regulated by leptin [54]. It is also tempting to speculate a role for leptin in HAT considering that disturbance in the circadian rhythm of the melatonin-generating systems in the pathogenesis of African sleeping sickness has been demonstrated [55].

Brucipain may also alter processes linked to arginine methylation. CARM1 is a protein arginine N-methlytransferase that plays a role in protein arginine methylation, a process that is implicated in signal transduction, nascent pre-RNA metabolism and transcriptional activation [56]. These data suggest that CARM1, as a promoter-specific regulator of NF-κB-dependent gene expression [57], could play a role in the inflammatory responses associated with CNS HAT.

In contrast to the Cluster-1 pathways, 3 hours exposure to the brucipain-inhibited trypanosomes upregulated 9 pathways represented in Cluster-8 (Figure 1, Table 1). Inhibiting brucipain activity appeared to activate the eukaryotic initiation factor-2 (EIF2)-pathway. EIF2 binding to GTP and Met-tRNA would in turn initiate translation by transferring the Met-tRNA to the 40S ribosomal subunit. If brucipain plays a role in downregulating this pathway, the event could shut-down cellular protein synthesis and could have consequences to overall cell viability [58].

The response to activation of PAR-2 is the elevation of intracellular Ca^2+^ via the PLC/IP_3_ pathway [59],[60], which leads to downstream increases in intracellular Ca^2+^, activation of PKC, mitogen-activated protein kinases (MAPK) and/or stress-activated protein kinases [61],[62],[63]. It has been also shown in other systems that direct PAR-2 activation of extracellular signal-regulated kinase (ERK) subfamily of MAPK [64] may be neuroprotective [47],[48],[49]. Because of the above characteristics associated with PAR-2 activation, it is remarkable that the most dramatic changes that happened between 3 and 6 hours in the pathways were associated with Cluster-11 and Cluster-15 (Table 1). Cluster-11 contained 4 pathways with strong positive Z-scores in response to the brucipain-inhibited trypanosomes. Of these 4 pathways, 3 were involved either with cell signaling (BREAST_CANCER_ESTROGEN_SIGNALING) or inflammatory responses (CLASSIC_PATHWAY, IL7_PATHWAY). In contrast to Cluster 11, all pathways in Cluster 15 displayed negative Z-scores, indicating downregulation. In some respects, Cluster 15 is the most interesting as it contained pathways that conceivably play a role in parasite transmigration of the BBB as an early event, and in the subsequent later inflammatory responses associated with HAT. The involvement of the ST_GAQ_PATHWAY (Fig. 4, Table 1) is interesting given that calcium signaling may play an important role in trypanosome / BBB associated events. Furthermore, the changes in the ST_GA13- SA_TRKA_RECEPTOR, ST_DICTOYOSTELIUM_DISCOIDEUM_CAMP_CHEMOTAXIS, and CELL_2_CELL-pathways also parallel roles for PI3K, MAPK and cell cytoskeleton. A role for brucipain as an inducer of inflammatory responses [4] is also predicted (i.e., EPO_NFKB_PATHWAY, RNA_PATHWAY).

### Conclusions

We studied parasite proteases in the interaction of *T. b. rhodesiense* with HBMECs. Overall, our RNAi, PMT and DNA-microarray data support an important role for brucipain, HBMEC PAR-2 (and possibly other PARs) and G_q_ signaling for trypanosome transmigration across the BBB. Owing to PAR-2's role in neuroinflammation, these data also suggest a role for this GPCR in CNS HAT. In murine models of HAT the neuroinflammatory response [1],[65] is likely a balance between pro-inflammatory cytokines such as interferon-γ (IFN-γ), interleukin-1(IL-1) and tumour necrosis factor-α (TNF-α), and counter-inflammatory cytokines such as IL-10 [66]. A role for cytokines in determining entry of trypanosomes into the CNS was provided by a seminal study in knockout mice in which the gene for IFN-γ had been disrupted [67]. Following systemic infection, it was found that trypanosomes accumulated in the perivascular regions, ‘trapped’ between the endothelial and the parenchymal basement membranes. While these findings suggested that lymphocyte-derived IFN-γ is required for trypanosome traversal across cerebral blood vessels [67], precisely how IFN-γ facilitates BBB traversal by the parasites has yet to be determined. Interestingly, minocycline, a tetracycline antibiotic, was also found to impede the penetration of leukocytes and trypanosomes into the brain parenchyma [68]. It is tempting to speculate a role for PAR-2 in these processes. It has been shown that the inflammatory response in mouse colonic tissue mediated by PAR-2 activation (using PAR-2 agonists) that leads to i) increased tissue permeability, ii) increased IFN-γ and TNF-α expression, and iv) decreased IL-10 expression, are abolished in IFN-γ deficient B6 mice [69]. More recently, minocycline has been shown to block the PAR-2-mediated TNF-α-induced production of IL-8 proinflammatory response in epidermal keratinocytes (Ishikawa) [70].

Our previous work strongly suggested that *T. b. rhodesiense* crosses the human BBB by generating Ca^2+^ activation signals in HBMECs through the activity of parasite cysteine proteases [5],[9]. Using *T. b. brucei* silenced for (RNAi) it was later found that parasite cathepsin L (brucipain) could be the parasite factor initiating transmigration and increased vascular permeability [11]. While a singular role has not been established for this process of parasite transmigration *in vivo*, GPCR PAR2 is one of the molecular targets for brucipain as an activator of Gq-mediated calcium-signaling involving the downstream effectors PLC and PKC [5],[9]. The *in vivo* consequence of these events, similar to our in vitro findings, is predicted to be increased permeability of the BBB to parasite transmigration, an event precursory to CNS disease [4],[11]. The critical role of brucipain raises the possibility for this protein as an attractive drug or vaccine target.

**Figure 5:**
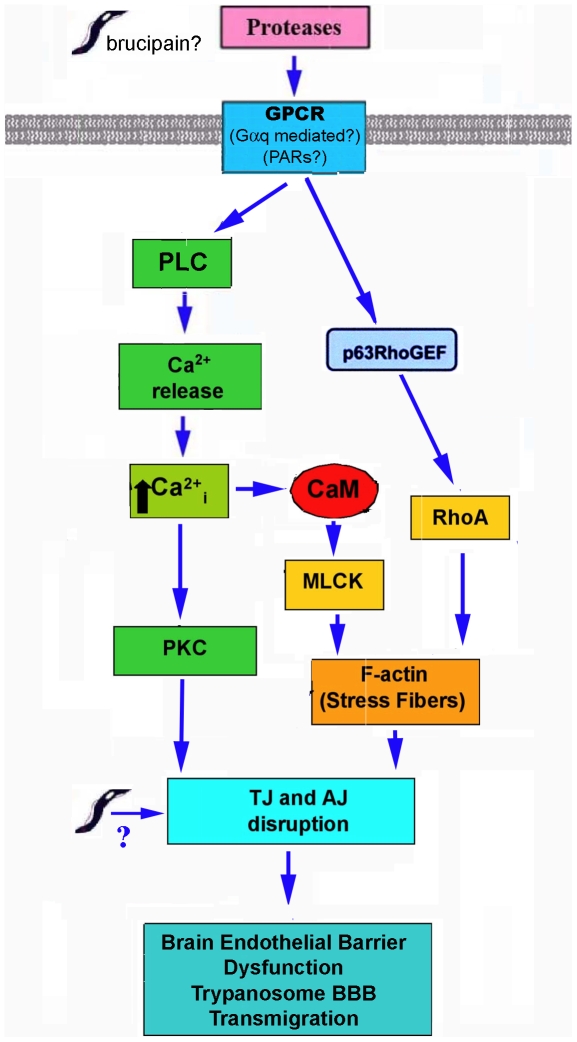
Proposed model for African trypanosome-induced BBB dysfunction. We hypothesize that parasite proteases trigger GPCRs (i.e. PARs?) via Gαq activation, which leads to PLC-mediated Ca^2+^ release from intracellular stores. The increase in intracellular calcium leads to calmodulin (CaM) activation of myosin light chain kinase (MLCK), ultimately leading to cytoskeletal changes and barrier dysfunction. Ca^2+^-independent activation of the cytoskeleton mediated by Ras-superfamily GTPases (i.e. RhoA) is also possible via p63RhoGEF. Parasite and/or host-derived proteases may also contribute by degrading or altering adherens junction (AJ) and tight junction (TJ) proteins.

Based on our published and findings reported here [4],[5],[9],[10], we hypothesize that African trypanosome-mediated BBB dysfunction is linked to the interactions of parasite and/or parasite protease activity with BMEC GPCRs (i.e. PARs) (Fig. 5). Activation of downstream effectors then enable African trypanosome transmigration through the BBB after cytoskeletal rearrangements that induce cell retraction and loosening of junctional complexes. We predict that trypanosomes/parasite/proteases trigger Gαq activation of PLC-β, in turn generating inositol-1,4,5-triphosphate (IP_3_) and diacylglycerol (DAG) from phosphatidylinositol-4,5-bisphosphate (PIP_2_). Binding of IP_3_ to its receptor on the endoplasmic reticulum releases Ca^2+^ from intracellular stores. The increase in intracellular calcium leads to calmodulin (CaM) activation of myosin light chain (MLC) kinase (MLCK) and/or other effectors ultimately leading to cytoskeletal changes and barrier dysfunction. Ca^2+^-independent activation of the cytoskeleton by Ras-superfamily GTPases (i.e RhoA) traditionally by Gα_12/13_ GPCRs, is also possible via Gαq activation of p63RhoGEF [42],[71]. Although not yet determined, parasite and/or host-derived proteases may also contribute by degrading or altering adherens junction (AJ) and tight junction (TJ) proteins.

Clearly, a study on the contribution of the products of gene expression identified with the biochemical pathways needs to be investigated as well as testing using established animal models of HAT [4]. An understanding of these responses at a molecular level will help identify candidates for the early diagnosis, treatment, and prevention of CNS invasion with HAT.
